# The Effect of Maropitant, Ondansetron and Metoclopramide on Dexmedetomidine‐Induced Vomiting in Cats

**DOI:** 10.1002/vms3.70152

**Published:** 2025-01-10

**Authors:** Ayşe Gölgeli Bedir, Latif Emrah Yanmaz, Sıtkıcan Okur, Mümin Gökhan Şenocak, Ferda Turgut, Yakup Kocaman, Ömer Tarık Orhun, Uğur Ersöz, Büşra Baykal

**Affiliations:** ^1^ Department of Surgery Faculty of Veterinary Medicine Atatürk University Erzurum Turkey; ^2^ Department of Surgery Faculty of Veterinary Medicine, Burdur Mehmet Akif Ersoy University Burdur Turkey; ^3^ Department of Surgery Faculty of Veterinary Medicine, Yozgat Bozok University Yozgat Turkey

**Keywords:** cats, dexmedetomidine, maropitant, metoclopramide, ondansetron, vomiting

## Abstract

**Objectives:**

Vomiting is frequently observed in cats after dexmedetomidine administration. This study aimed to compare the efficacy of different antiemetics in preventing vomiting in cats after dexmedetomidine administration.

**Methods:**

Sixty‐four cats were randomly allocated to receive saline solution (0.9% NaCl, 0.1 mL/kg, SC), maropitant (1 mg/kg, SC), ondansetron (0.22 mg/kg, IM) or metoclopramide (1 mg/kg, IM) 30 min before the intramuscular administration of dexmedetomidine (25 µg/kg). Duration of vomiting, severity of vomiting and nausea signs (sialorrhoea, lip licking, retching and vomiting) was recorded for 30 min after dexmedetomidine administration.

**Results:**

The duration and severity of vomiting were significantly reduced in groups that received maropitant, ondansetron or metoclopramide compared to the saline group. Although differences were observed in retching and vomiting between the saline and other groups (*p* < 0.001), there were no significant differences in sialorrhoea or lip licking (*p* = 0.34 and *p* = 0.12, respectively).

**Conclusions:**

Maropitant, ondansetron and metoclopramide were found to significantly reduce retching and vomiting compared to the control group. In conclusion, no significant difference was found among maropitant, ondansetron and metoclopramide groups in the prevention of dexmedetomidine‐induced vomiting in cats.

## Graphical abstract

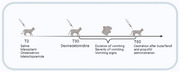

## 1 Introduction

Nausea in cats is commonly associated with vomiting, salivation, lip licking, restlessness, excessive swallowing, abnormal body posture and lethargy (Kenward et al. 2015; Elliot 2019). During the perioperative period, vomiting in animals can negatively impact their comfort level and lead to complications, such as aspiration pneumonia, gastroesophageal reflux and esophagitis (Hall, Clarke, and Trim 2001). Moreover, vomiting can result in serious complications such as increased intraocular or intracranial pressure (Papastefanou et al. 2015).

Dexmedetomidine is commonly utilized in clinical applications for sedation and premedication in cats, either administered alone or in combination with other sedative drugs (McSweeney et al. 2012; Granholm et al. 2006). Following intramuscular administration, dexmedetomidine induces vomiting in the majority of cats (Slingsby, Taylor, and Monroe 2009; Santos et al. 2011; Nagore et al. 2013). Dexmedetomidine's sedative effects are mediated by α_2_‐receptor activation in the locus coeruleus, the brain's largest noradrenergic cell group (Scheinin and Schwinn 1992), whereas its emetic effects arise from α_2_‐receptor activation in the postrema region of the chemoreceptor trigger zone (Colby, McCarthy, and Borison 1981; Duke et al. 1994).

Various anti‐emetics have been evaluated to assess their impact on the vomiting reflex induced by α_2_ adrenergic drugs (Kolahian 2014, Santos et al. 2011, Papastefanou et al. 2015, Martin‐Flores et al. 2016b, 2016a, 2017). Santos et al. (2011) suggested that ondansetron administration reduced the severity of vomiting in cats with dexmedetomidine‐induced vomiting. Another study demonstrated that metoclopramide effectively reduces xylazine‐induced vomiting (Kolahian et al. 2014). A study evaluating the antiemetic effects of butorphanol reported that it reduces the incidence and severity of dexmedetomidine‐induced vomiting in cats (Papastefanou et al. 2015). Previous studies have shown that the administration of maropitant at various doses reduces the incidence of vomiting in cats induced by dexmedetomidine and morphine (Martin‐Flores et al. 2016b, 2016a, 2017). However, to date, no study has compared the effects of maropitant, ondansetron and metoclopramide on dexmedetomidine‐induced vomiting in cats. Thus, the objective of this study was to evaluate the effects of maropitant, ondansetron and metoclopramide on the incidence, frequency and severity of vomiting in cats after dexmedetomidine administration. We hypothesize that metoclopramide, ondansetron and maropitant would similarly reduce the incidence of vomiting following intramuscular administration of dexmedetomidine in cats.

## 2 Materials and Methods

### 2.1 Animals

A total of 64 client‐owned cats were admitted to the Atatürk University Animal Hospital for elective castration. The study only included cats that had a normal complete blood count, and serum chemistry, and were classed as American Society of Anesthesiologists (ASA) physical status I. Cats were disqualified if they exhibited symptoms of illness, had irregular heart rhythms, were under 6 months old or beyond 3 years old, or had an ASA status higher than I. Cats that had a history of nausea or diarrhoea or who had evidence of sialorrhoea, abdominal pain, haematochezia or halitosis on physical exam were excluded from the study. The day before the study, all cats were admitted to the cat ward for acclimatization. Food was withheld for 6 h before the procedure. Water was always available. All experimental procedures took place between 8:00 AM and 11:00 AM. The cats were discharged from the hospital the following day.

### 2.2 Study Design

**FIGURE 1:**
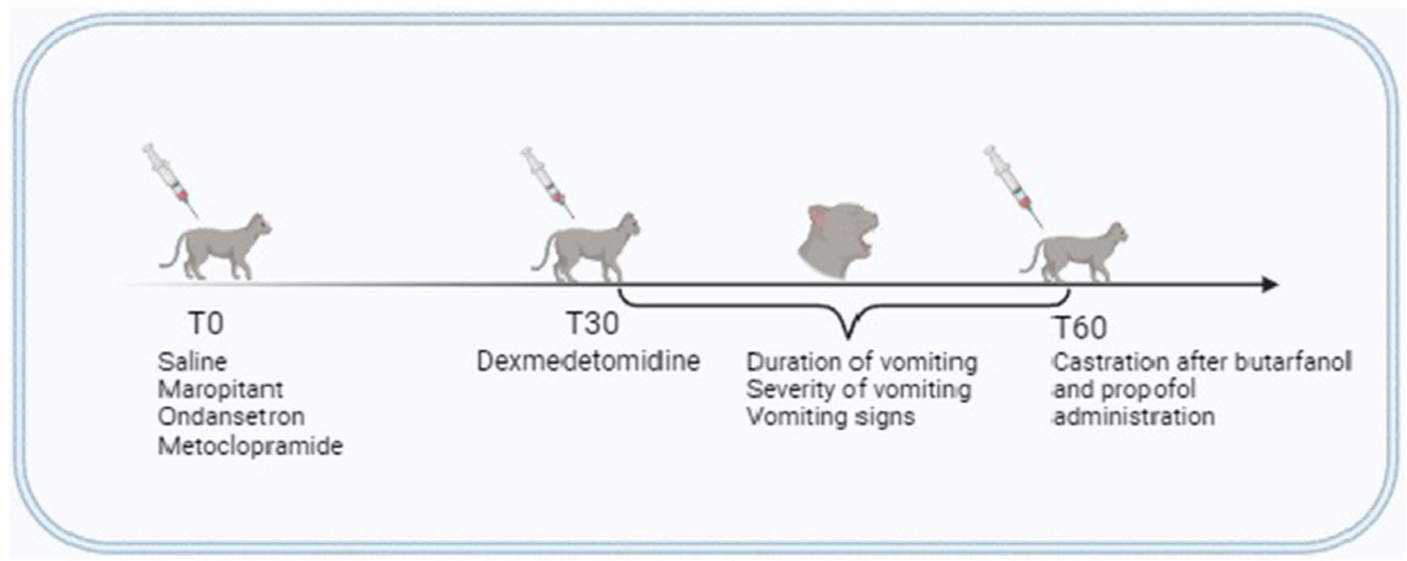
Evaluation of vomiting in cats administered saline solution (0.9% NaCl, 0.1 mL/kg, SC, *n* = 16), maropitant (1 mg/kg, SC, *n* = 16), ondansetron (0.22 mg/kg, IM, *n* = 16) or metoclopramide (1 mg/kg, IM, *n* = 16) 30 min before dexmedetomidine (25 µg/kg, IM).

Cats were randomly assigned to receive saline (0.9% NaCl) solution (0.1 mL/kg, SC; saline group; *n* = 16), maropitant^a^ (1 mg/kg, SC; maropitant group; *n* = 16), ondansetron^b^ (0.22 mg/kg, IM; ondansetron group; *n* = 16) or metoclorpramide^c^ (1 mg/kg, IM; metoclopramide group; *n* = 16) 30 min before IM administration of dexmedetomidine^d^ (25 µg/kg). All injections were administered by a single individual, who was unaware of the study details, using a 1 mL syringe with a 22G needle, delivering a total dose of 1 mL. Administered intramuscularly, injections were targeted specifically at the quadriceps muscle of the left hind leg. Physiological parameters were measured in the following sequence: heart rate (HR), respiratory rate (*f*_R_), rectal temperature (RT) and mean arterial pressure (MAP). HR was evaluated using a stethoscope, *f*_R_ was calculated by observing movements in the chest, RT was recorded using a digital thermometer inserted 4 cm into the rectum and MAP was measured using an electronic oscillometric device (petMap; Ramsey Medical) with the cuff placed near the carpus. Vomiting severity and frequency were independently assessed by two observers who were unaware of the treatment administered while the cats were in their cages. The frequency and severity of vomiting were monitored for 30 min following the administration of dexmedetomidine. After this period, all animals were given subcutaneous butorphanol^e^ (0.2 mg/kg) and then intravenous propofol^f^ (1 mg/kg). Postoperative analgesia was primarily provided by dexmedetomidine, with butorphanol administered subcutaneously as a mild adjunctive analgesic and sedative. No further observations were made at this stage, and the castration procedure was performed (Figure 1).

### 2.3 Vomiting Score Evaluation

Vomiting, retching, sialorrhoea and lip licking were monitored in all cats during the study. The severity of vomiting was scored as mild (1), moderate (2) or severe (3), as previously described by De La Puente‐Redondo et al. (2007). Specifically, mild vomiting was characterized by nonproductive retching, moderate vomiting involved vomiting without bile and severe vomiting included vomiting containing bile. During a 30‐min observation period following IM dexmedetomidine injection, the number of vomiting episodes and the duration of vomiting were recorded for each cat. The duration of vomiting in cats was measured by calculating the time each instance of retching and vomiting lasted in seconds (Gralla et al. 1981).

Retching is characterized by the coordinated contraction of the abdominal and diaphragm muscles, without the actual expulsion of stomach contents. On the other hand, vomiting refers to the vigorous expulsion of stomach contents through the mouth (Scholz, Steinfath, and Tonner 2004). Signs of nausea were considered present when sialorrhoea (defined as the collection of clear or frothy fluid around the lips with or without dripping) and excessive licking of the lips were observed (Santos et al. 2011).

### 2.4 Statistical Analysis

A power analysis was performed to ascertain the minimal sample size needed. In order to reach a research power of 95% with a significance level of 0.05, a minimum of 16 cats was required to detect a 5% difference in the incidence of nausea between the saline and anti‐emetic medication groups. All cats enrolled in the study were included in the analysis. The data were analysed with an exclusive software application (Statistic 9.0 Analytical Software, Tallahassee, Florida, USA). Power calculations were based on previous prospective studies using dexmedetomidine in cats (Granholm et al. 2006; Monteiro et al. 2009; Slingsby, Taylor, and Monroe 2009) and our observations from the shelter's spay program the previous year. Fisher's exact test (which is suitable for yes/no [vomited/did not] data with small group size) was used for double‐checking the power calculations for pairs of treatments. Because several continuous variables had a standard deviation that was 50% of their respective means, a nonparametric statistical analysis was employed. Due to the presence of three distinct treatment groups, a Kruskal–Wallis test was applied to compare the frequency of emetic events, duration until the first emetic event and the severity of vomiting. Where a significant difference was detected, a Wilcoxon rank‐sum test was used to identify any specific differences among groups.

## 3 Results

The cats were an average age of 12.2 ± 3.8 months and weighed 3.2 ± 0.5 kg, with no significant differences between groups. The cats in this study included British shorthair (24), Mixed (30) and Scottish shorthair (10) breeds.

**FIGURE 2:**
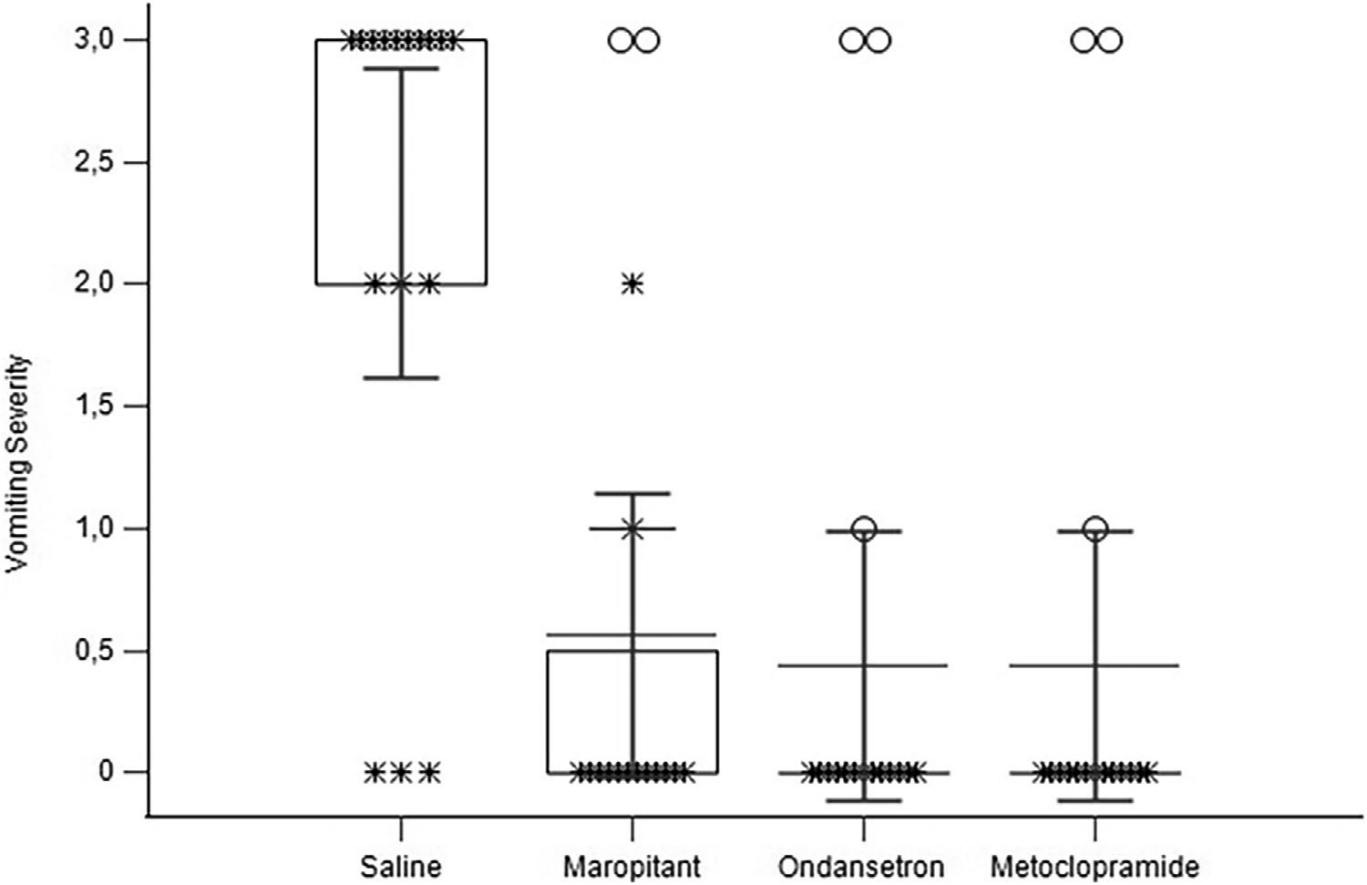
The severity of vomiting in cats administered either saline solution (0.9% NaCl, 0.1 mL/kg, SC, *n* = 16), maropitant (1 mg/kg, SC, *n* = 16), ondansetron (0.22 mg/kg, IM, *n* = 16) or metoclopramide (1 mg/kg, IM, *n* = 16) 30 min before dexmedetomidine (25 µg/kg, IM) administration is illustrated as follows: Asterisks indicate the severity of vomiting in each cat within the groups, whereas circles denote cats with distinct values in each group.

In the saline group, 68% of the cats experienced vomiting, with each vomiting episode occurring only once per cat. After IM injection of dexmedetomidine, the duration of vomiting was significantly decreased in the maropitant, ondansetron and metoclopramide groups compared with the saline group (maropitant vs. control *p* = 0.0001, ondansetron vs. control *p* = 0.0001, metoclopramide vs. control *p* = 0.0001). The severity of vomiting was also significantly reduced in these treatment groups compared to the saline group (*p* < 0.001). No significant differences in the severity or incidence of vomiting were observed among the maropitant, ondansetron and metoclopramide groups (maropitant vs. ondansetron *p* = 0.088, maropitant vs. metoclopramide *p* = 0.122 and metoclopramide vs. ondansetron *p* = 0.91; Figure 2).

Retching and vomiting were observed in 81.2% and 68.7% of the saline group. In contrast, in the maropitant group, they were observed in 25% and 18.8%, in the ondansetron group in 18.8% and 12.5%, and in the metoclopramide group in 18.8% and 12.5%, respectively. Although significant differences were found between the saline and other groups for retching and vomiting (*p* < 0.001), there were no significant differences between the saline and other groups for sialorrhoea, or lip licking (*p* = 0.34 and *p* = 0.12, respectively). In this study, maropitant, ondansetron and metoclopramide significantly reduced retching and vomiting compared with the control group (Table 1).

**TABLE 1:** The incidences of sialorrhoea, lip licking, retching and vomiting were observed in cats that were administered either saline solution (0.9% NaCl, 0.1 mL/kg, SC, *n* = 16), maropitant (1 mg/kg, SC, *n* = 16), ondansetron (0.22 mg/kg, IM, *n* = 16) or metoclopramide (1 mg/kg, IM, *n* = 16) 30 min before dexmedetomidine (25 µg/kg, IM).

	Signs of nausea			
Group	Sialorrhoea (%)	Lip licking (%)	Retching (%)	Vomiting (%)
**Saline**	14/16 (87.5)	14/16 (87.5)	13/16 (81.2)^a^	11/16 (68.7)^a^
**Maropitant**	13/16 (81.2)	11/16 (68.7)	4/16(25)^b^	3/16 (18.8)^b^
**Ondansetron**	10/16 (62.5)	8/16 (50)	3/16 (18.8)^b^	2/16 (12.5)^b^
**Metoclopramide**	11/16 (68.7)	9/16 (56.2)	3/16 (18.8)^b^	2/16 (12.5)^b^

*Note*: Different letters in each column indicate significant differences among groups (*p* < 0.001).

## 4 Discussion

In this study, the administration of maropitant, ondansetron and metoclopramide significantly decreased the incidence of vomiting in cats following the intramuscular administration of 25 mcg/kg dexmedetomidine in cats.

In a previous study, dexmedetomidine was reported to cause 81% vomiting in cats after IM administration at a dose of 7 µg/kg (Thawley and Drobatz 2015). Santos et al. (2011) observed vomiting in 78% of cats in the group without ondansetron after administering dexmedetomidine at a dose of 40 µg/kg IM. In a study by Papastefanou et al. (2015) that induced vomiting in cats using different doses of dexmedetomidine, it was observed that in the groups not receiving butorphanol, the vomiting rates were 71% for the 20 µg/kg IM dose and 78% for the 25 µg/kg IM dose. Similarly, in our study, the administration of dexmedetomidine at a dose of 25 µg/kg IM in cats resulted in a vomiting rate of 68.7% in the saline group.

The effectiveness of antiemetic drugs in reducing the duration of vomiting depends on various factors, including the underlying cause of the symptoms, the type of antiemetic used and individual patient characteristics (Shaikh et al. 2016). This is the first study to evaluate the duration of vomiting in cats. In a study evaluating the effectiveness of ondansetron and metoclopramide in preventing postoperative nausea in humans, the antiemetic drug used decreased the duration of vomiting (Morris et al. 1998). Another study on humans who received chemotherapy demonstrated that metoclopramide has significantly shorter durations of vomiting than saline solution (Gralla et al. 1981). Similarly, the present study indicated that maropitant, ondansetron and metoclopramide decrease the duration of vomiting compared to saline.

Regarding sialorrhoea and lip licking, no significant differences occurred in this study among all groups. However, the incidence of retching and vomiting was significantly lower in maropitant, ondansetron and metoclopramide compared to the saline group. Martin‐Flores et al. (2016b) found that maropitant (1 mg/kg) administered intramuscularly 20 h prior to dexmedetomidine (20 µg/kg) and morphine (0.1 mg/kg) reduced the incidence of vomiting and retching in cats compared to the saline group, whereas the incidence of sialorrhoea and lip licking remained unchanged. Martin‐Flores et al. (2016b) suggested in their study that this might be because, although sialorrhoea and lip licking are associated with nausea, it is more challenging to perceive these signs in veterinary patients. Martin‐Flores et al. (2017) reported that administering an oral dose of 8 mg of maropitant to cats 2–2.5 h before intramuscular administration of dexmedetomidine and morphine did not reduce the incidence of sialorrhoea and lip licking in either the maropitant‐treated cats or the antiemetic‐free (control group) cats. They suggested that the lack of change in the incidence of sialorrhoea and lip licking might be due to the maropitant's lack of direct effect on the vomiting mechanisms of dexmedetomidine and morphine (Martin‐Flores 2017). The results of this study are in line with results of previous studies in cats (Martin‐Flores et al. 2016b, Martin‐Flores et al. 2017).

The medications used in our study demonstrate their effects on vomiting through different mechanisms (Kolahian 2014; Corrêa et al. 2019; Kolahian and Jarolmasjed 2010; Thompson and Lummis 2007). The chemoreceptor trigger zone in cats is highly enriched with α_2_‐adrenoreceptors, and the effect of dexmedetomidine on vomiting is related to the activation of these receptors (Kolahian 2014). Maropitant is a neurokinin‐1 receptor antagonist and exerts its antiemetic effect through this mechanism (Corrêa et al. 2019). Metoclopramide exerts an antiemetic effect by antagonizing dopamine and serotonin receptors, and it prevents dex‐induced vomiting by inhibiting these receptors in the bilateral nucleus tractus solitarii along the nervous pathway (Kolahian and Jarolmasjed 2010). Ondansetron exerts its antiemetic effect by antagonizing serotonin receptors in the area postrema (Thompson and Lummis 2007). As a result, one of the reasons maropitant, ondansetron and metoclopramide may not control all signs of nausea is that these drugs do not target the α_2_ adrenergic receptors.

There are some limitations in our study, the first being the potential for sex bias due to the lack of female cats, as suggested by Salazar‐Parra et al. (2020), who found that nausea and vomiting were more common in women following laparoscopic cholecystectomy. Moreover, a previous study evaluating the prevalence of vomiting in young dogs reported a higher incidence in male dogs (Martin‐Flores et al. 2016a). The second limitation is the inability to assess the antiemetic effect of the drugs over a longer period. However, it is worth noting that nausea has been observed within the first 15 min after dexmedetomidine administration (Papastefanou et al. 2015, Santos 2011, Slingsby and Taylor 2008). The third limitation is that evaluating sialorrhoea and lip licking as indicators of nausea in cats is difficult and subjective.

## 5 Conclusion

Maropitant, ondansetron and metoclopramide exhibit similar efficacy in preventing dexmedetomidine‐induced vomiting in cats, both in terms of frequency and severity. This provides veterinarians with flexibility in choosing the most appropriate antiemetic based on factors like cost, availability, and potential side effects.

## Author Contributions

All the authors contributed to the study design, material preparation, data collection, statistical analysis, supervision and writing of the manuscript and approved the final manuscript.

## Ethics Statement

The Atatürk University (Decision number: 169) Local Board of Ethics Committee for Animal Experiments approved the experimental protocol.

## Conflicts of Interest

The authors declare no conflicts of interest.

### Peer Review

The peer review history for this article is available at https://publons.com/publon/10.1002/vms3.70152.

## Data Availability

The datasets generated during and/or analysed during the current study are available from the corresponding author upon reasonable request.

## Endnotes

a Maropitant; Cerenia, Pfizer Animal Health, New York, NY.

b Ondansetron; Osetron, Haver Farma, İstanbul, Turkey.

c Metoclopramide; Anti‐Nausea, Onfarma, Samsun, Turkey.

d Dexmedetomidine; Dexdomitor, Pfizer Animal Health Inc., NY, USA.

e Butorphanol; Butomidor, Richter Pharma, Austria.

f Propofol; Propofol‐ PF, Polifarma, Tekirdağ, Turkey.
